# Supplementary material for: Improving Lurasidone Hydrochloride’s Solubility and Stability by Higher-Order Complex Formation with Hydroxypropyl-β-cyclodextrin
Source: Pharmaceutics. 2023 Jan 10;15(1):232. doi: 10.3390/pharmaceutics15010232 (PMC9861442; doi:10.3390/pharmaceutics15010232)
Supplement: Supplementary file 1 [file pharmaceutics-15-00232-s001.zip › pharmaceutics-2057955-Supplementary Materials.pdf]

pharmaceutics

MDPI

Supplementary Material: Improving Lurasidone Hydrochloride's Solubility and Stability by Higher-Order Complex Formation with Hydroxypropyl-β-cyclodextrin

María Elena Gamboa-Arancibia, Nelson Caro, Alexander Gamboa, Javier Octavio Morales, Jorge Enrique González Casanova, Diana Marcela Rojas Gómez and Sebastián Miranda-Rojas

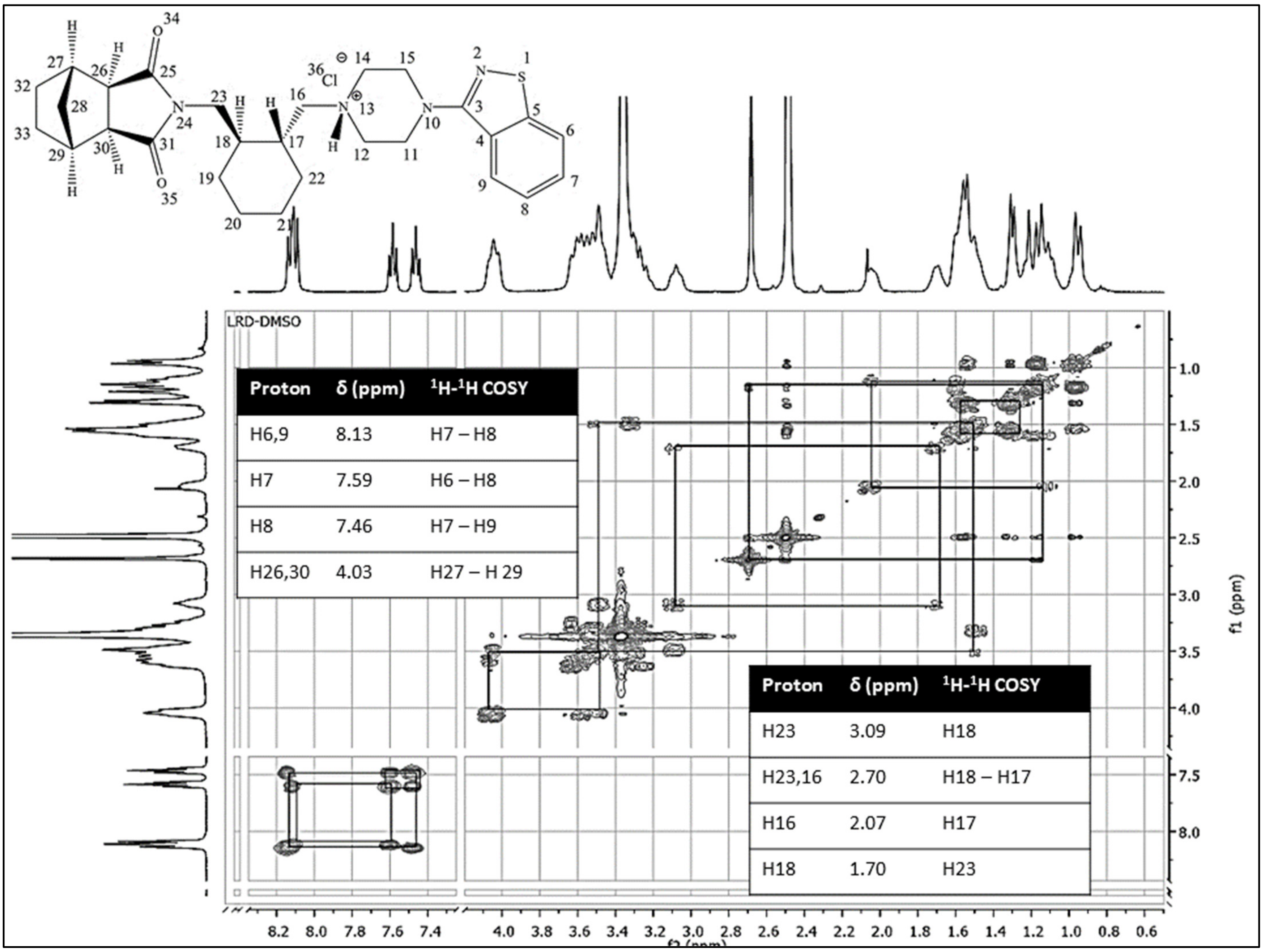

Figure S1. ^{1}H-^{1}H correlation spectroscopy experiment of LRD.

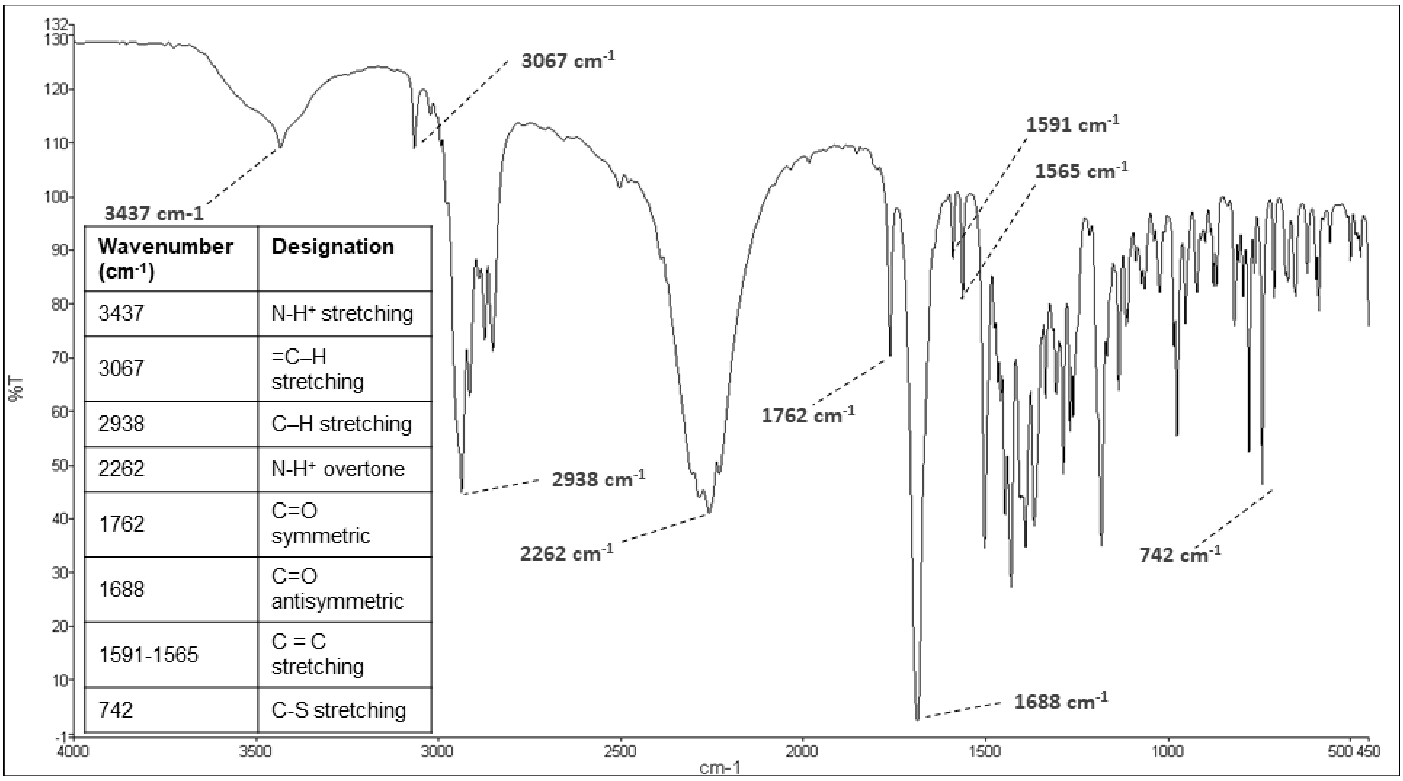

Wavenumber (cm-1)	Designation
3437	N-H+ stretching
3067	=C–H stretching
2938	C–H stretching
2262	N-H+ overtone
1762	C=O symmetric
1688	C=O antisymmetric
1591-1565	C = C stretching
742	C-S stretching

Figure S2. Infrared spectrum of LRD.

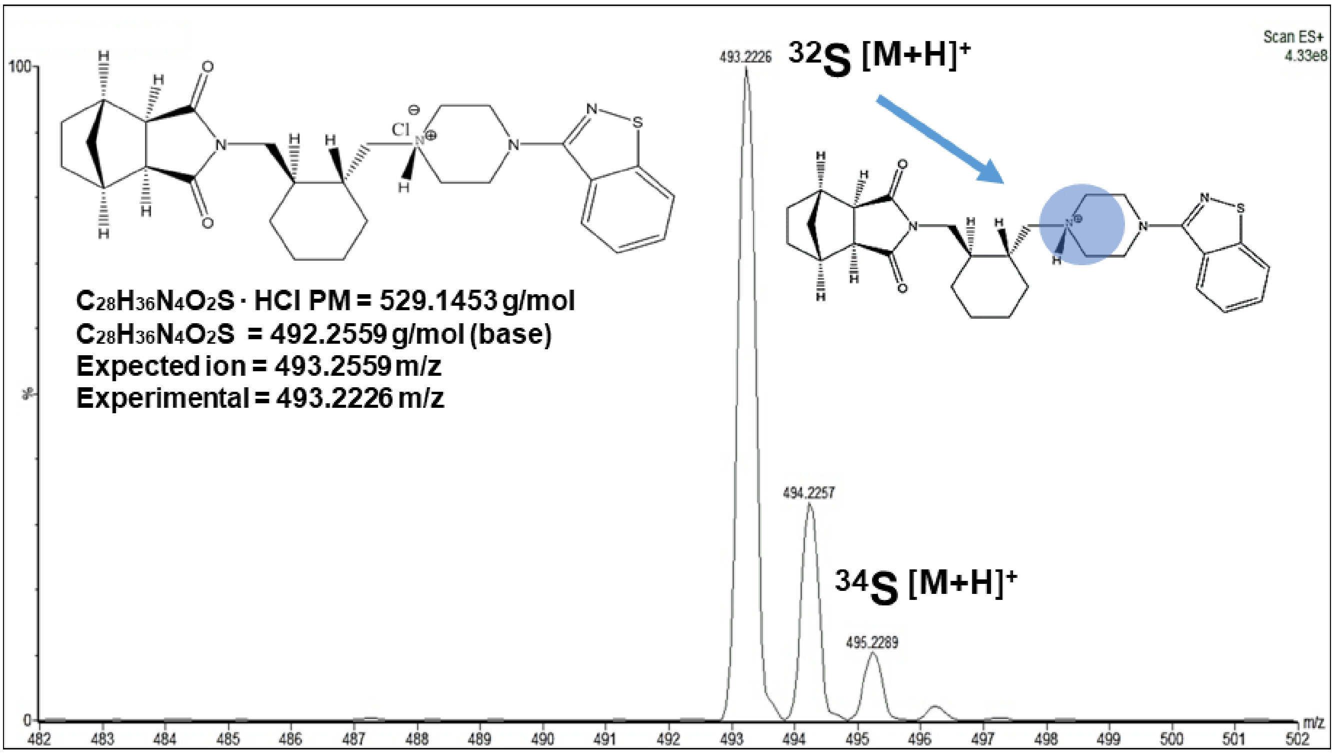

Figure S3. Mass spectrum of LRD.

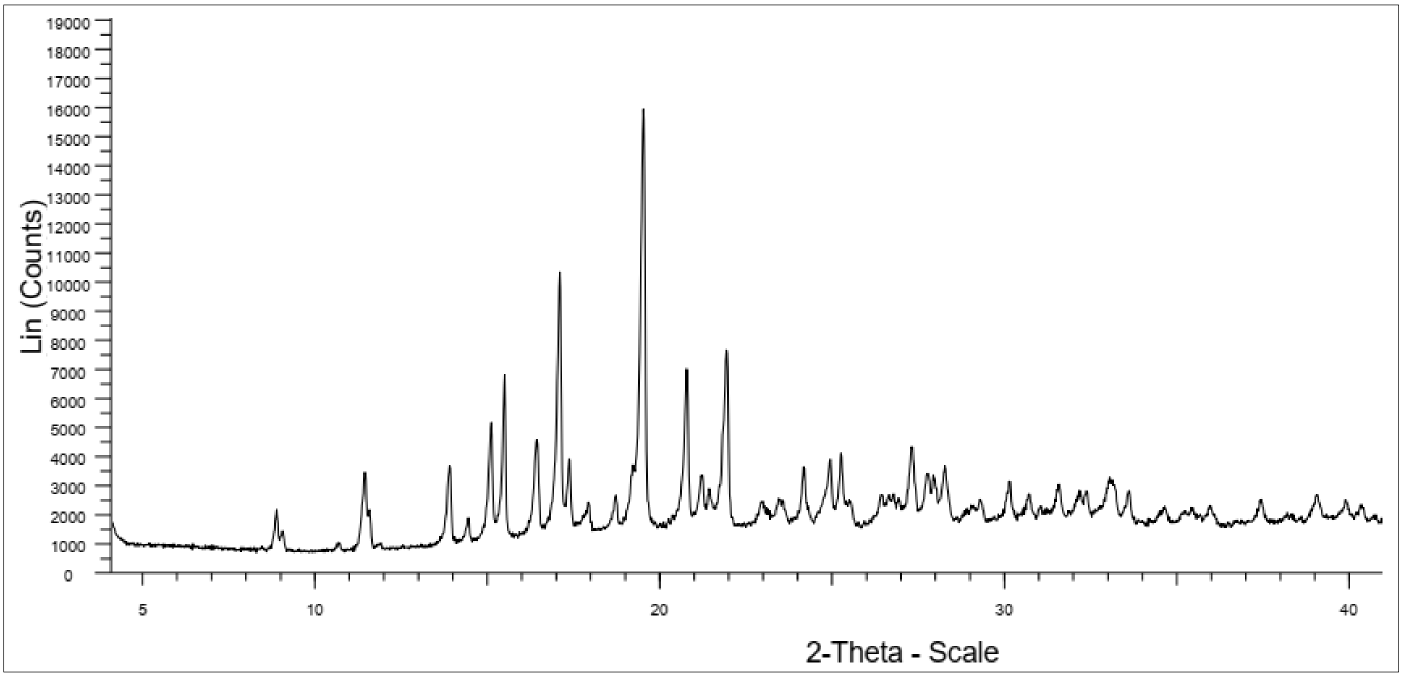

Figure S4. Powder X-ray diffraction of LRD.

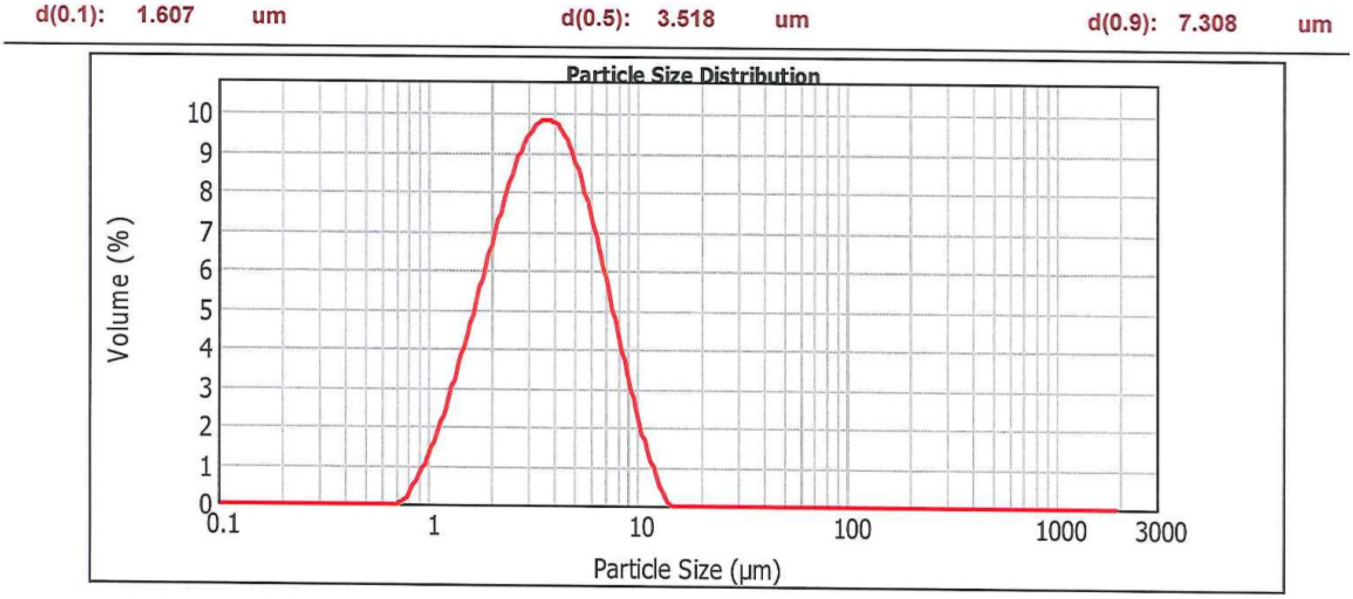

Figure S5. Particle size distribution of LRD.

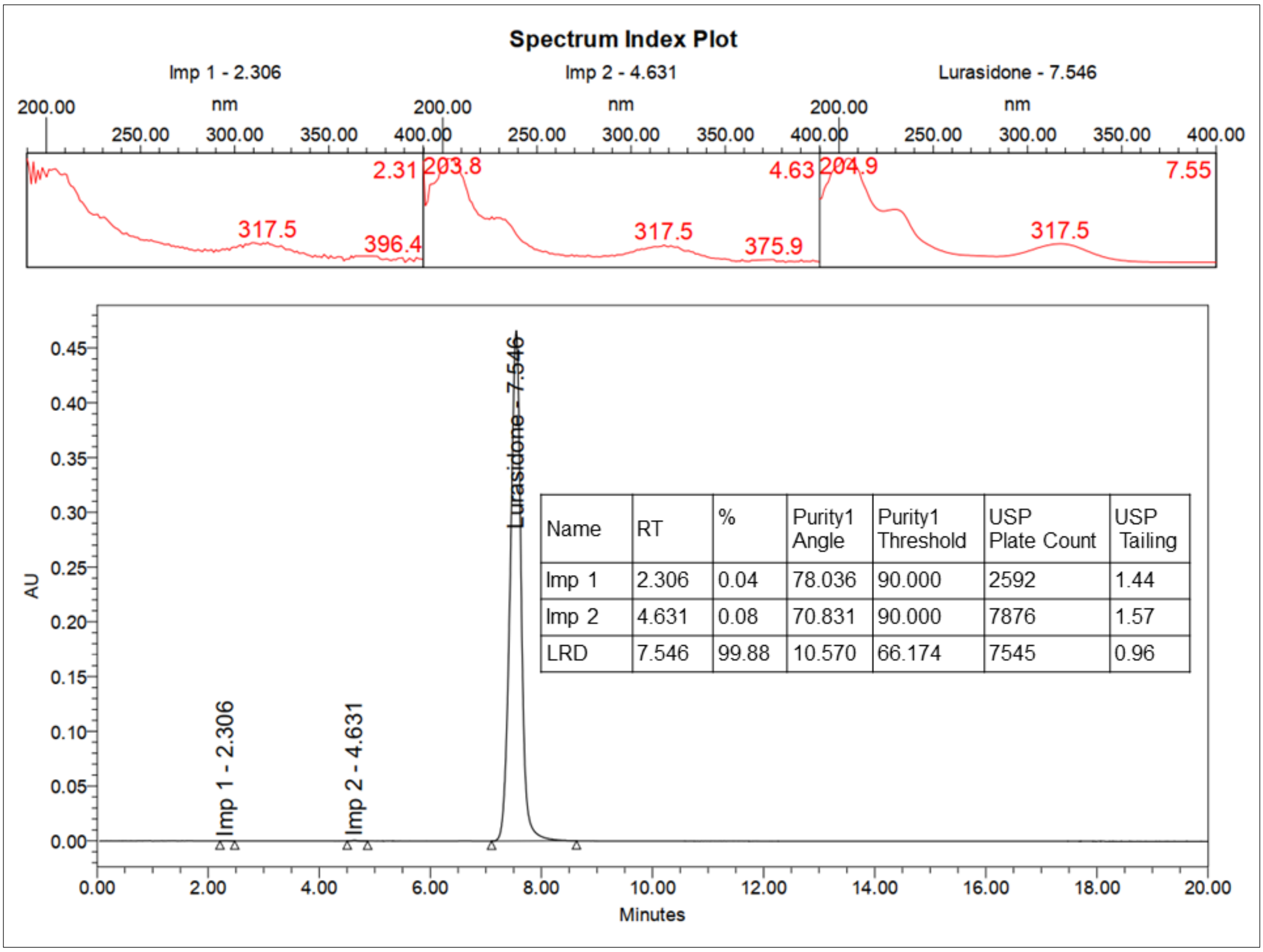

Figure S6. HPLC purity of LRD.

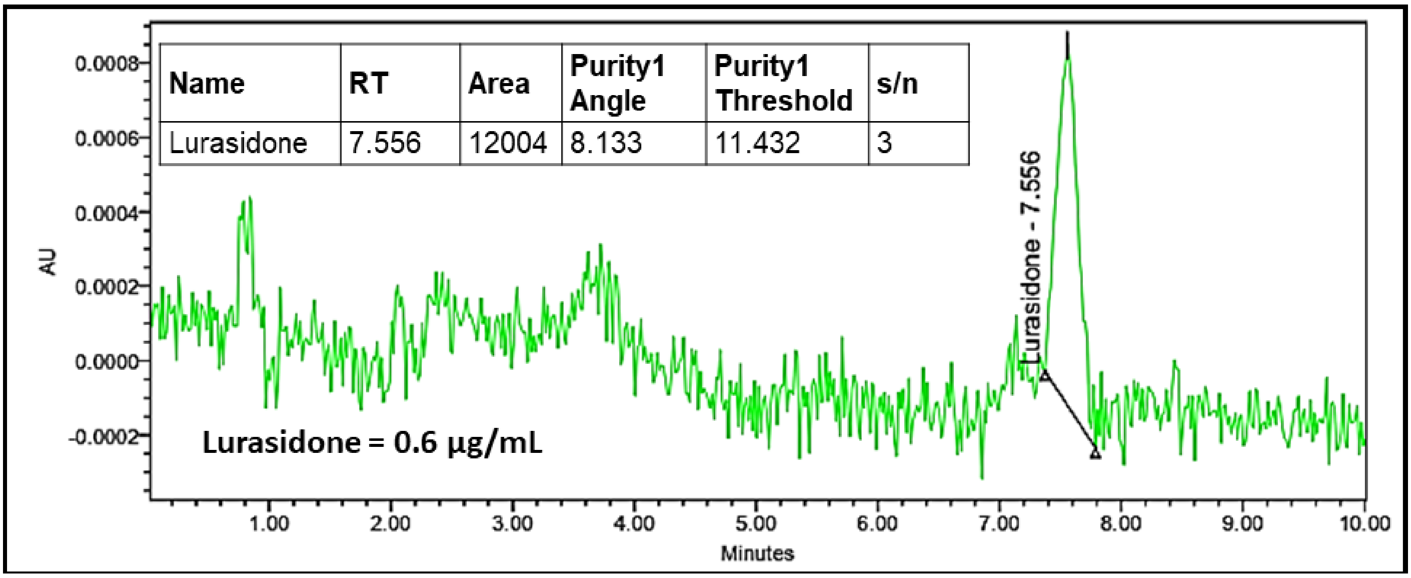

Figure S7. Chromatogram of the detection limit

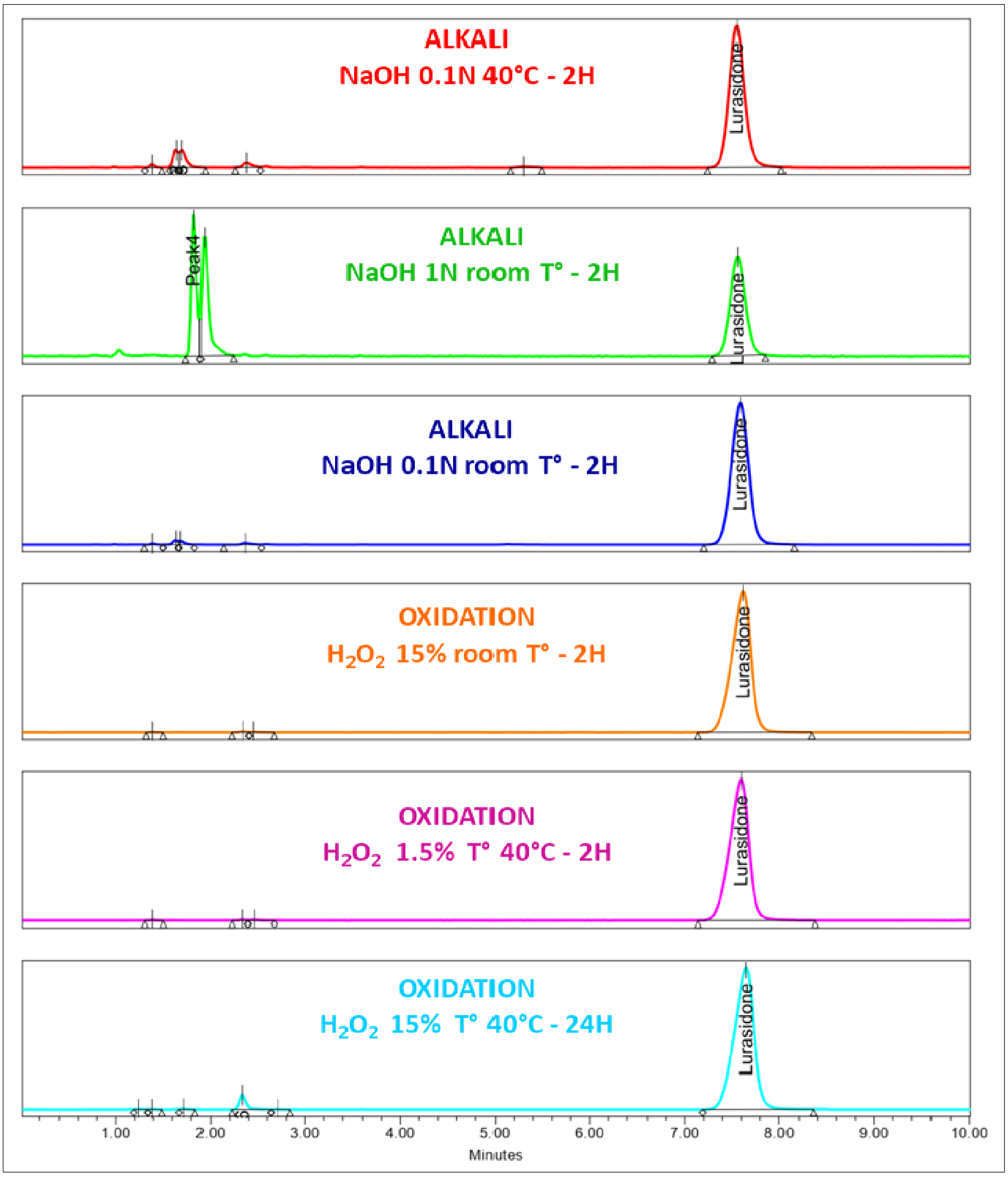

Figure S8. Chromatograms of the LRD degradation in the evaluation of the specificity of the method.

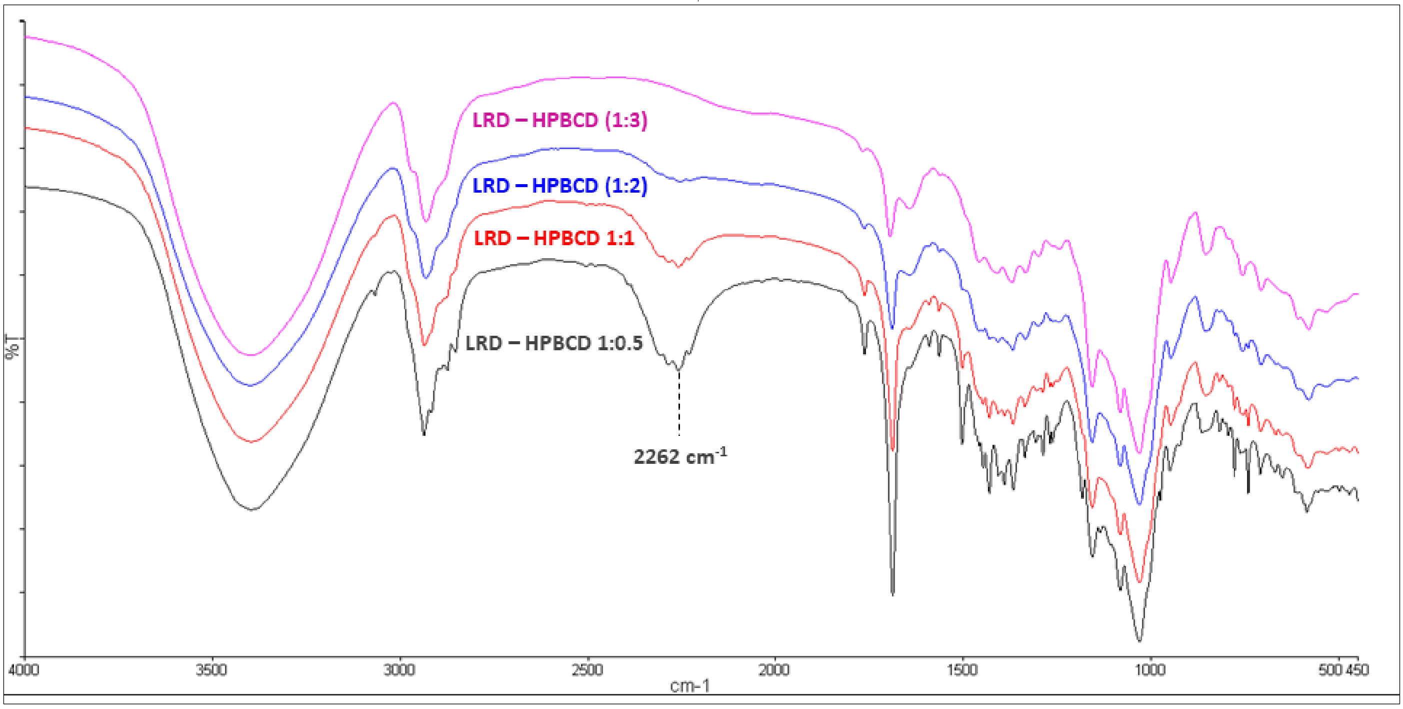

Figure S9. Infrared spectroscopy analysis of LRD-HPBCD complexes at 3 months accelerated stability.

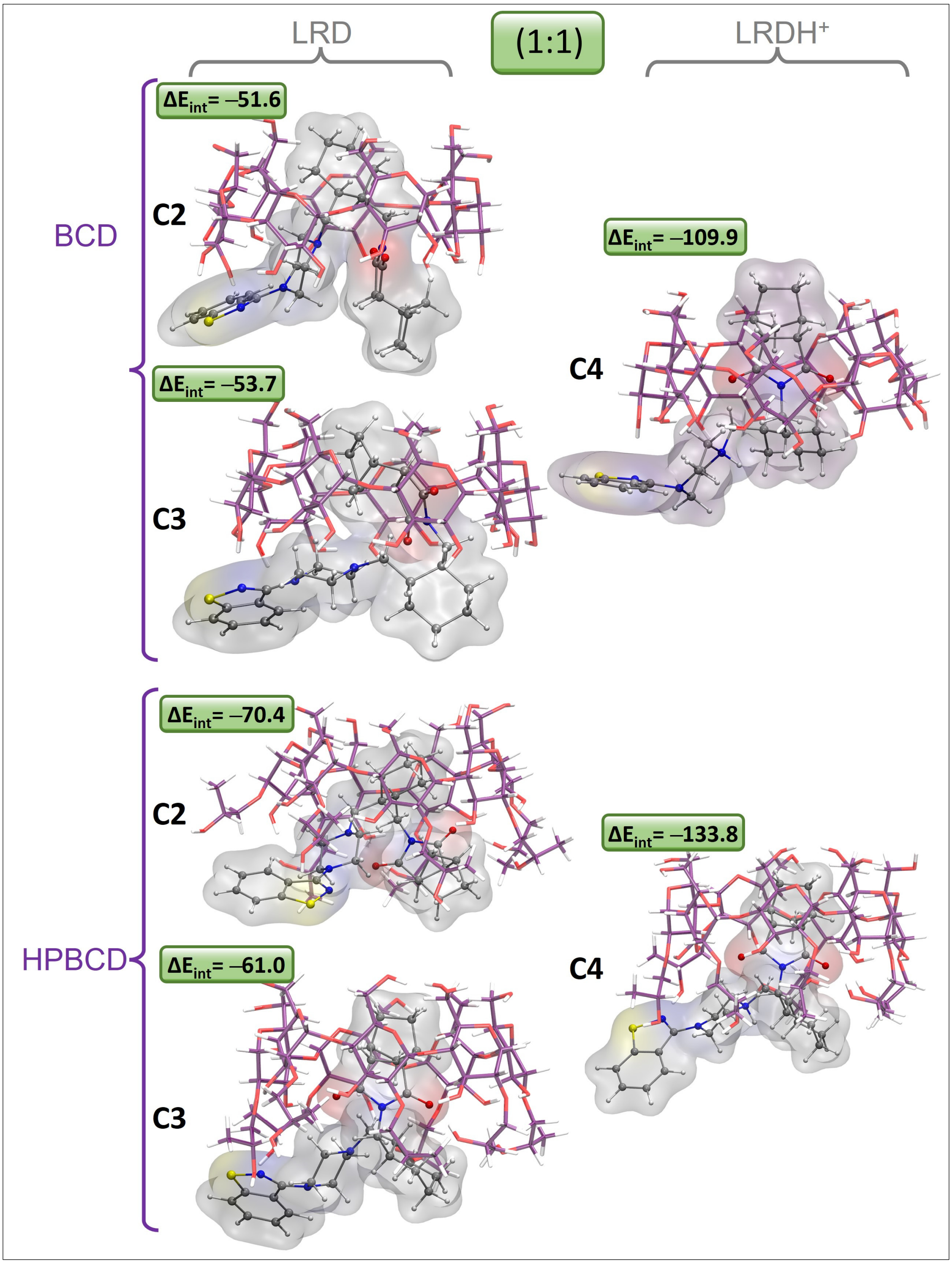

Figure S10. Graphical representation of the rest of the binding conformations found for the inclusion complexes for LRD and LRDH into BCD and HPBCD for the first inclusion process (1:1) (cyclodextrin carbon: purple, LRD/LRDH carbon: gray, hydrogen: white, oxygen: red, nitrogen: blue, sulfur: yellow; ΔE_{int} stands for interaction energy in kcal/mol).

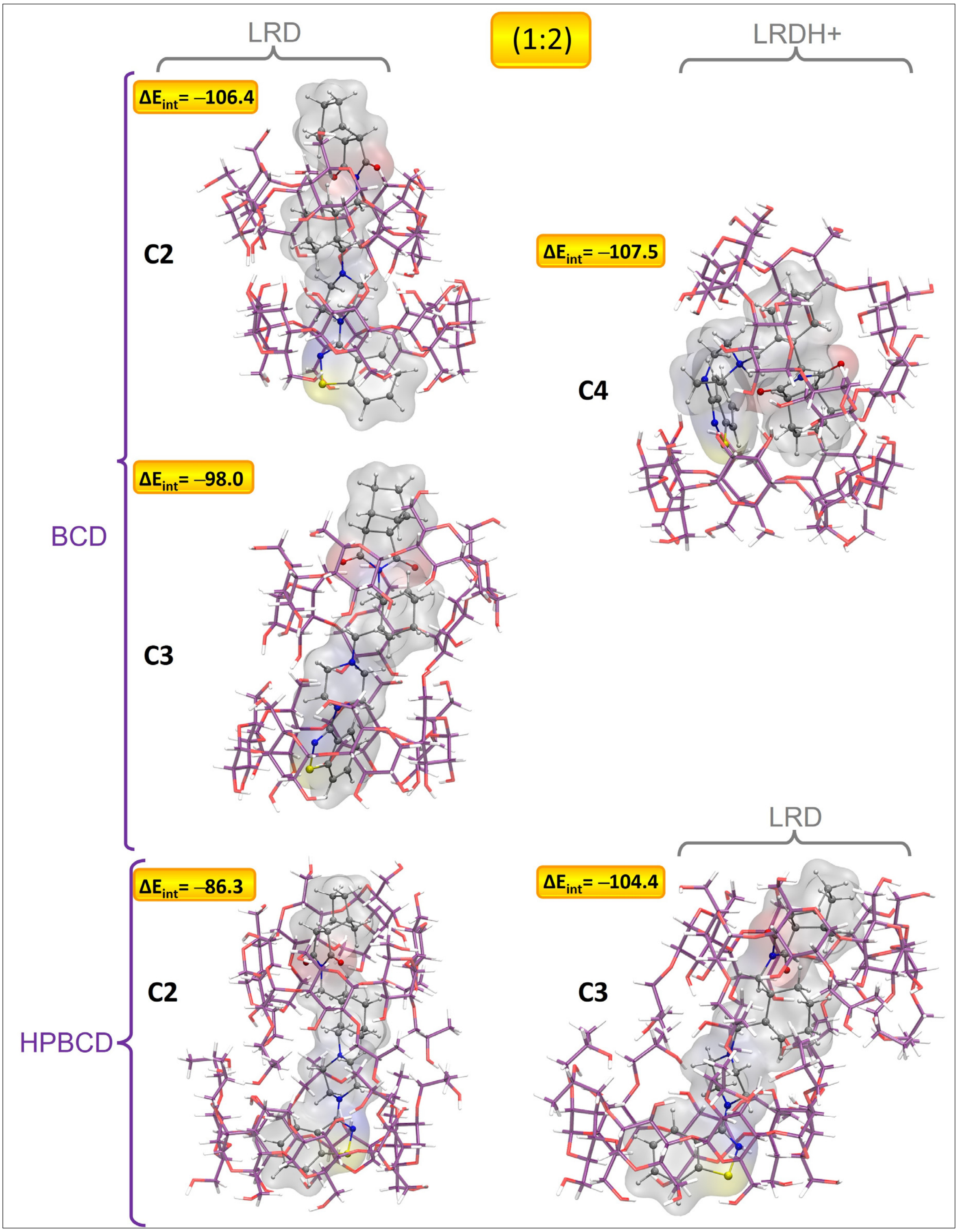

Figure S11. Graphical representation of the geometries representation of the rest of the binding conformations found for the inclusion complexes for LRD and LRDH into BCD and HPBCD for the second inclusion process (1:2) (cyclodextrin carbon: purple, LRD/LRDH carbon: gray, hydrogen: white, oxygen: red, nitrogen: blue, sulfur: yellow; ΔE_{int} stands for interaction energy in kcal/mol).

Table S1. Interaction energies (ΔE_{int}), interaction energy corrected by implicit solvent ($\Delta E_{int\text{-}S}$), solvent destabilization contribution (Solv), dispersion interaction energy contribution (E_{disp}), and dipolar moments (μ) for the complex formation between lurasidone and BCD or HPBCD in a (1:1) and (1:2) stoichiometry. Energies are in kcal/mol and μ is in debye (D).

Complex	ΔE_{int}	$\Delta E_{int\text{-}S}$	Solv %	μ [a]	E_{disp}
(1:1)					
LRD-BCD-C2	-51.6	-36.7	28.9	9.55	-72.6
LRD-BCD-C3	-53.7	-42.7	20.5	10.83	-73.4
LRDH$^+$-BCD-C4	-109.9	-92.8	15.6	15.78	-61.4
LRD-HPBCD-C2	-70.4	-55.6	21.0	14.86	-82.5
LRD-HPBCD-C3	-61.0	-53.6	12.1	8.27	-73.5
LRDH$^+$-HPBCD-C4	-133.8	-104.4	22.0	19.62	-68.5
(1:2)					
LRD-BCD-C2	-106.4	-78.0	26.7	9.55	-82.4
LRD-BCD-C3	-98.0	-69.4	29.2	12.78	-63.6
LRDH$^+$-BCD-C4	-107.5	-93.8	12.7	12.20	-54.8
LRD-HPBCD-C2	-86.3	-61.5	28.7	10.52	-72.8
LRD-HPBCD-C3	-104.4	-77.9	25.4	6.99	-100.8

[a] The reference dipolar moments of LRD in its neutral and charged forms are 3.45 D and 8.15 D.
